# A novel biochemical analysis for ApoE4 quantification in plasma and discrimination of homozygous and heterozygous *APOE* ε4 carriers

**DOI:** 10.1186/s13195-025-01811-w

**Published:** 2025-07-15

**Authors:** Andrés Rodríguez, Olga Calero, Sergio Veiga, Miriam Menacho-Román, Ignacio Arribas, Lluís Cano, Guillermo García-Ribas, Miguel Calero

**Affiliations:** 1 Biocross S.L, Valladolid, Spain; 2 Chronic Disease Programme (UFIEC), Instituto de Salud Carlos III, Madrid, Spain; 3 Centro de Investigación Biomédica en Red de Enfermedades Neurodegenerativas (CIBERNED), Instituto de Salud Carlos III, Madrid, Spain; 4 Service of Clinical Biochemistry, Ramón y Cajal University Hospital, Madrid, Spain; 5 Institute Ramón y Cajal for Health Research (IRYCIS), Madrid, Spain; 6 Linear Chemicals, S.L.U, Montgat, Barcelona, Spain; 7 Department of Neurology, Hospital Universitario Ramón y Cajal, Madrid, Spain

**Keywords:** Apolipoprotein E4 (ApoE4), Alzheimer's disease (AD), Biochemical assay, Plasma biomarkers, Non-genetic test, Amyloid-related imaging abnormalities (ARIA), Personalized medicine, Turbidimetric immunoassay, *APOE* genotyping alternative

## Abstract

**Background:**

The *APOE* ε4 allele is the strongest genetic risk factor for late-onset Alzheimer’s disease (AD) and is associated with increased risk of amyloid-related imaging abnormalities (ARIA) during anti-amyloid therapy. Accurate identification of ε4 carriers, particularly *APOE* ε4/ε4 individuals, is clinically relevant. This study outlines the development and validation of e4Quant, a novel turbidimetric assay for quantifying plasma ApoE4 as a non-genetic alternative to *APOE* genotyping.

**Methods:**

The e4Quant test utilizes a proprietary particle-enhanced immunoturbidimetry method, employing an isoform-specific anti-ApoE4 antibody on standard chemistry analyzers. Plasma samples from 160 individuals of known *APOE* genotype (35 *APOE* ε4/ε4 homozygotes, 115 *APOE* ε4 heterozygotes, and 10 *APOE* ε4 non-carriers) were analyzed for ApoE4 and total ApoE levels. The test’s discriminatory performance was assessed by ROC analysis and two-threshold classification algorithms.

**Results:**

ApoE4 levels ascertained by the e4Quant test exhibited clear genotype-dependent stratification. ROC analysis indicated 100% sensitivity and specificity in distinguishing *APOE* ε4 carriers from non-carriers, and 88.6% sensitivity and 90.4% specificity for discriminating homozygous from heterozygous carriers. Normalizing ApoE4 to total ApoE improved classification (sensitivity 94.3%, specificity 93.9%). A combined ratio-plus-concentration approach further enhanced discrimination (sensitivity 91.4%, specificity 100%). Three ε4 homozygous samples with low ApoE4/total ApoE ratios were misclassified.

**Discussion:**

The e4Quant assay offers a rapid, cost-effective, and highly accurate biochemical alternative to *APOE* genotyping, suitable for clinical and research settings, particularly in assessing AD risk and optimizing anti-amyloid therapeutic strategies. One subgroup of *APOE* ε4/ε4 subjects had unexpectedly low ApoE4 levels, raising questions about potential biological heterogeneity and its impact on Alzheimer’s disease biology.

**Conclusion:**

The e4Quant assay is a novel alternative for genotyping to determine *APOE* ε4 carrier status, while also providing quantitative measurements of ApoE4 levels. Its high diagnostic accuracy, ease of use in standard clinical laboratories, and potential utility for personalized medicine in AD treatment highlight its translational value. Further studies are warranted to investigate the clinical significance of *APOE* ε4 expression variability and its impact on disease progression and treatment response.

**Supplementary Information:**

The online version contains supplementary material available at 10.1186/s13195-025-01811-w.

## Background

Apolipoprotein E (ApoE) is a 34 kDa glycoprotein essential for lipid metabolism. Initially, its relevance was linked to its role in regulating plasma lipoprotein levels and atherogenesis; however, its significance expanded with the discovery of its strong association with neurodegenerative diseases, particularly Alzheimer’s disease (AD). The human *APOE* gene is polymorphic, with three distinct alleles (ε2, ε3, and ε4) that correspond to three different ApoE isoforms (ApoE2, ApoE3, and ApoE4) [1]. These isoforms differ only at amino acids 130 and 176 (corresponding to positions 112 and 158, respectively, when numbering does not include the 18-residue signal peptide). Isoform E2 has cysteine at both sites, E4 has arginine at both sites, while the most common form, E3, has a cysteine at position 130 and an arginine at position 176.

*APOE* ε4 is present in approximately 20% of the global population and 40–60% of AD patients [2–6]. The presence of one ε4 allele of the *APOE* gene increases the risk of AD by 3–5 times, while homozygous carriers have an 8–12 times increase [7]. Thus, the ApoE4 isoform is the most potent genetic risk factor for sporadic AD. Moreover, *APOE* ε4 carriers progress more rapidly from mild cognitive impairment (MCI) to Alzheimer’s disease dementia than non-carriers [8], although the precise pathophysiology of this risk remains unclear. Also, carriers of one, and especially two copies of the ε4 allele, show an increased risk of cerebral amyloidosis and cognitive dysfunction earlier than expected [9].

In addition to AD risk, *APOE* ε4 carriers, especially those who are *APOE* ε4/ε4 homozygotes, exhibit an increased susceptibility to Amyloid-Related Imaging Abnormalities (ARIA) when treated with anti-amyloid antibodies [10, 11]. Notably, ARIA-E (ARIA with edema/effusion) incidence is significantly higher in *APOE* ε4 homozygous individuals receiving amyloid-lowering therapies [12, 13]. Thus, determining the *APOE* ε4 status is increasingly important in both clinical and research settings, as it informs disease risk stratification, therapeutic eligibility, patient safety monitoring, and contributes to biomarker and epidemiological research.

Interestingly, population-based studies in the oldest old (those 90 years and older) have reported a lower prevalence of *APOE* ε4 compared to younger populations. Moreover, on average, centenarians are twice as likely as the general population to carry an *APOE* ε2 allele [14–16]. Although *APOE* ε2 provides a protective effect against AD, it is also linked to lipid homeostasis disorders, including familial type III hyperlipoproteinemia [17, 18].

Given its clinical and research significance, *APOE* genotyping remains the gold standard for *APOE* ε4 status analysis in epidemiological, molecular, and clinical studies. Various genetic methods are commonly used for genotyping the three major *APOE* haplotypes. These include DNA sequencing, specifically labeled DNA probes, real-time PCR, PCR followed by restriction enzyme digestion, and many others [19–21]. While these *APOE* genotyping methods are highly effective, they require DNA extraction, which limits their accessibility in general laboratory settings.

Alternatively, biochemical (protein-based) methods can directly detect ApoE isoforms in biological fluids, such as plasma or cerebrospinal fluid (CSF) [22–25]. Recently, as alternatives to genetic testing, a study evaluated two plasma-based proteotyping assays: a Chemiluminescence Enzyme Immunoassay (CLEIA) developed by Fujirebio (Fujirebio Diagnostics Japan, Inc., Japan) and a prototype NULISA (Nucleic Acid–Linked Immunosandwich Assay) method [26]. Both methods enable genotype classification based on protein levels but require a pair of antibodies to capture and detect the ApoE4 isoforms. They are also constrained by complexity, cost, or platform exclusivity, which limits their widespread implementation in clinical laboratories.

We have developed e4Quant (Biocross SL), a quantitative refinement of the CE-marked e4Risk^®^ turbidimetric test (Biocross SL) [27, 28]. The e4Quant assay enables a quantitative estimation of plasma ApoE4, the sensitive differentiation of *APOE* ε4 carriers from non-carriers, and the detection of *APOE* ε4/ε4 homozygotes. Unlike conventional ELISA or CLEIA, the e4Quant assay is a rapid and cost-effective method that relies on the specific binding of ApoE to polystyrene beads [27], thereby precluding the need for a capture antibody or prior separation procedures. This method is easily implemented in biochemistry labs within general hospitals. Its simplicity, compatibility with standard lab infrastructure, and capacity to deliver quantitative ApoE4 data add clear value in personalized AD risk assessment, treatment planning, and inclusion in prevention trials. In this report, we present a validation of e4Quant, comparing its performance to genetic *APOE* testing in the same individuals.

## Methods

### Test description and reagent composition

The Biocross e4Quant assay is a test for ApoE4 quantitation in plasma based on a particle-enhanced immunoturbidimetric assay (PETIA), a technique widely used by chemistry analyzers already available in hospitals and clinical laboratories. The test is composed of two ready-to-use reagents (R1 and R2), a set of four calibrators, and high and low controls that serve as quality controls for the assay. The R1 reagent is provided as a solution containing an anti-ApoE4 antibody (4E4 clone with reference #NBP1-49529 from Novus Biologicals (Abingdon, UK)), while the R2 reagent is a suspension containing polystyrene particles (latex, reference #AJ20COOH-L2, IKERLAT polymers SL (Gipuzkoa, Spain)). Calibrators are prepared from human plasmas from *APOE* ε4 non-carriers (Diaserve Laboratories GmbH (Iffeldorf, Germany)), spiked with human recombinant ApoE4 (Ref. #350-04-500UG from Peprotech (London, UK), at present distributed by ThermoFisher Scientific (Waltham, Massachusetts, US)). High and low controls consist of a pool of plasmas from *APOE* ε4 carriers and *APOE* ε4 non-carriers (Diaserve Laboratories GmbH, Iffeldorf, Germany). Calibrators and controls are provided as lyophilized powder.

### Validation across platforms

Verification was performed at two independent clinical laboratories to ensure the accuracy and adaptability of the e4Quant assay across different automated high-throughput clinical chemistry analyzers. At the University Hospital Ramón y Cajal in Madrid, Spain, the assay was tested on an Atellica (Siemens) analyzer. At the *Hospital Universitario La Paz* in Madrid, Spain, it was tested on an Alinity (Abbott) analyzer. No modifications were made to platform-specific configuration parameters, and calibration was performed using the e4Quant calibrator set. Calibration acceptance required that the coefficient of variation (CV) for absorbance measurements across four calibrators, each run in triplicate, did not exceed 10% (see Supplementary Table 1).

A set of low- and high-control samples with known ApoE4 concentrations was included to confirm the accuracy of the measurement. Acceptance criteria required that the measured values remain within ± 20% of the expected concentration (see Supplementary Table 2).

A pre-genotyped set of samples was analyzed to assess the consistency of ApoE4 quantification between platforms. This dataset included five *APOE* ε4 non-carriers and three *APOE* ε4 carrier samples. A comparison of ApoE4 measurements across Atellica (Siemens) and Alinity (Abbott) platforms is shown in Supplementary Table 3.

### Plasma samples and *APOE* genotyping

The study cohort was established from a total of 160 EDTA plasma samples, distributed in 35 *APOE* ε4/ε4 homozygous, 115 *APOE* ε4 heterozygous (ε2/ε4 n = 6 and ε3/ε4 n = 109), and 10 *APOE* ε4 non-carriers (ε2/ε3 n = 3 and ε3/ε3 n = 7) individuals that were provided by Fundación ACE within the context of the project entitled *“Estudio de correlación de la concentración plasmática de ApoE4 y la concentración de Aβ42*,* Tau y p-Tau en el líquido cefalorraquídeo” (“Correlation study of plasma ApoE4 concentration and cerebrospinal fluid levels of Aβ42*,* Tau*,* and p-Tau”)* (Code: MED-FACE-2020-03). The Clinical Investigation Ethics Committee approved the study at Santa Creu i Sant Pau Hospital, Barcelona, Spain. This cohort is not representative of the general population; instead, it was intentionally enriched for *APOE* ε4 carriers, particularly in those who are ε4/ε4 homozygotes. This design choice was made to enable robust evaluation of the e4Quant test performance across different genotypes, particularly its ability to discriminate ε4 homozygotes from heterozygotes.

*APOE* genotypes were assessed using TaqMan genotyping assays for the rs429358 and rs7412 single-nucleotide polymorphisms (SNPs) (Thermo Fisher Scientific). Genotypes were furthermore extracted from the Axiom 815 K Spanish Biobank Array (Thermo Fisher Scientific), which was performed by the Spanish National Center for Genotyping (CeGen).

All samples were visually inspected prior to analysis. No abnormalities, such as hemolysis, lipemia, or turbidity, were observed in any of the samples included in this study.

### ApoE4 analysis

ApoE4 concentrations were determined using the e4Quant quantitative turbidimetric assay on the KROMA PLUS chemistry analyzer (Linear Chemicals S.L.U., Barcelona; throughput: 250 tests/hour). The assay is based on the European patent EP3274725 B1: “Method for Apolipoprotein Detection.” The analyzer was calibrated using a spline curve method with purified water as the 0 µg/mL point and a four-point calibrator set containing ApoE4 at known concentrations: Cal 01 (4.6 µg/mL), Cal 02 (8.2 µg/mL), Cal 03 (17.2 µg/mL), and Cal 04 (34.9 µg/mL). Each run included low (9.7 µg/mL) and high (19.2 µg/mL) quality controls. All calibrators were traceable to a Biocross-developed gold standard based on lyophilized plasma from an *APOE* ε3/ε4 individual, with ApoE4 levels quantified by mass spectrometry [29, 30]. For each reaction, 5 µL of calibrator, control, or plasma sample was mixed with 110 µL of reagent R1 and 110 µL of reagent R2. After gentle homogenization, initial absorbance at 546 nm (A1) was recorded immediately. Samples were then incubated at 37 °C for 5 min before the final absorbance (A2) was measured. The concentration of ApoE4 was calculated by interpolating the change in absorbance (A2– A1) on the calibration curve. Calibrators were analyzed in triplicate, while quality controls and plasma samples were run in duplicate to ensure reproducibility.

### Total ApoE analysis

Total ApoE concentration was determined using a commercial ApoE test (Spinreact, ref: 93014) on the chemistry analyzer KROMA PLUS (Linear Chemicals S.L.U., Barcelona) according to the manufacturer’s instructions. Shortly, the analyzer was calibrated with an APO-CAL (Spinreact, ref: 93005) (4.4 mg/dL) and purified water as 0 mg/dL. Each calibration point was analyzed in triplicate. Samples were analyzed in 2 rounds using the kit APO_CONTROL (Spinreact, ref: 93006) (3.5 mg/dL) as a quality control in each run. Concentration values from total ApoE controls were within the established range (2.7–4.0 mg/dL).

### Analysis of binding affinity of ApoE4 to polystyrene and detection

To explore the apparent binding affinity and detection of ApoE4 to blocked polystyrene surfaces used in turbidimetric assay, we used standard ELISA plates, which share similar material properties. In brief, polystyrene 96-well plates ELISA plates (Nunc Maxisorb flat-bottom 96 well plate) were blocked with a BSA-based blocking solution (0.25% BSA solution in 15 mM borate buffer containing 0.05% polysorbate 20 and 100 mM NaCl, pH 8.5). Plates were then washed with TBS-T (25mM Tris containing 0.1% polysorbate 20 and 150 mM NaCl, pH 7.4), before the addition of decreasing serial dilutions (50 to 2 nM) of recombinant ApoE4 diluted in TBS-T for 2 h at RT. Following incubation, plates were washed and incubated for 1 h at RT with either rabbit polyclonal pan-apoE antibody (H-223 from SantaCruz at 1:1,000 dilution) or the 4E4 monoclonal antibody specific for apoE4 (Novus Biologicals, #NBP1-49529, diluted 1:2,000) in TBS containing 20% of blocking solution for 1 h at RT. After washing, appropriate HRP-conjugated antibodies (anti-mouse IgG-HRP (#I1904-06 C) or anti-rabbit IgG-HRP (#I1904-40 A)) from US Biologicals were added at 1:10,000 dilutions in TBS-T plus 20% blocking solution for 30 min at RT. After washing, signal was developed using TMB chromogenic substrate (BioRad, #1721066) for 15 min at RT, stopped with 2.15 N sulfuric acid, and absorbance was measured at 450 nm with reference at 750 nm.

### Statistical analyses

All statistical analyses were performed using IBM SPSS Statistics software v25. Continuous variables, including ApoE4 concentration and total ApoE levels, were assessed for distributional assumptions. As data did not meet the criteria for normality or homogeneity of variances, non-parametric methods were applied to explore differences among groups.

Comparisons of ApoE4 and total ApoE levels among the three *APOE* genotype groups (non-carriers, ε4 heterozygotes, and ε4 homozygotes) were conducted using the Kruskal–Wallis one-way ANOVA test (k samples) with post hoc pairwise comparisons with Bonferroni correction to control for multiple testing.

To assess the discriminative performance of the e4Quant test, Receiver Operating Characteristic (ROC) curve analysis was employed. The Area Under the Curve (AUC) was calculated to evaluate the ability of ApoE4 concentration, total ApoE, and the ApoE4/total ApoE ratio to distinguish between genotype categories. Sensitivity, specificity, and optimal cut-off values were derived using the ROC curve data. A dual cut-off approach was used to define a high-specificity zone for homozygotes and a high-sensitivity zone for non-carriers, establishing a diagnostic grey zone where additional tests may be required.

Additionally, to evaluate the discriminatory power of the e4Quant assay across *APOE* genotypes, plasma samples were analyzed according to the measured ApoE4 concentration (Y-axis, µg/mL) and the ApoE4/total ApoE ratio (X-axis) (Fig. 3). Based on visual inspection of the data distribution, we generated several regions where the different *APOE* genotypes cluster (non-carriers, ε4 heterozygotes, and ε4 homozygotes). For this analysis, we also applied a two-threshold model classification by using parallel linear equations. Thus, two cut-off lines were defined as upper and lower boundaries that effectively divided the biomarker space into three informative zones: ε4 non-carriers (samples below the lower boundary), ε4 heterozygotes (samples between the boundaries), and ε4 homozygotes (samples above the upper boundary) (Fig. 3).

**Fig. 1:**
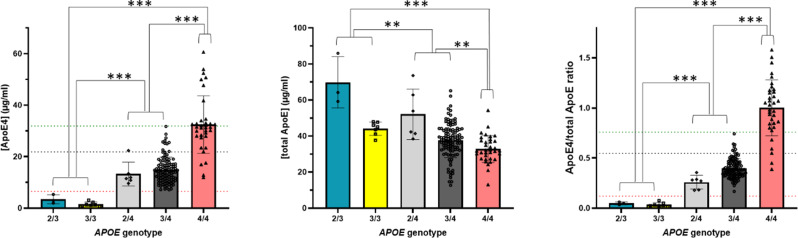
Plasma levels of ApoE4, total ApoE, and the ApoE4/total ApoE ratio across different *APOE* genotypes. Concentrations of ApoE4 (left), total ApoE (center), and the ApoE4/total ApoE ratio are shown for individuals stratified by *APOE* genotype. Each dot represents an individual sample; bars indicate mean ± SD. In the left panel, red dashed line marks the reference cut-off to discriminate *APOE* ε4-carriers from non-carriers (6.5 µg/ml), the black dashed line indicates the optimal threshold for distinguishing ε4 heterozygotes from ε4/ε4 homozygotes (21.8 µg/ml; specificity = 0.904; sensitivity = 0.886), and green dashed line indicates the high specificity threshold for ruling out heterozygotes from homozygotes (31.9 µg/ml; specificity = 1; sensitivity = 0.457). In the right panel, equivalent thresholds are shown for the ApoE4/total ApoE ratio: red dashed line for ε4-carriers vs. non-carriers discrimination (ratio = 0.12), black dashed line for optimal separation of heterozygotes from homozygotes (ratio = 0.548; specificity = 0.939; sensitivity = 0.943), and green dashed line for high-specificity threshold for ruling out heterozygotes from homozygotes (ratio = 0.759; specificity = 1; sensitivity = 0.857). Statistically significant differences for all pairwise comparisons were estimated by the Kruskal–Wallis one-way ANOVA test (k samples) with Bonferroni correction for multiple testing. Asterisks indicate level of significance: **p-values < 0.01; *** p-values < 0.001

**Fig. 2:**
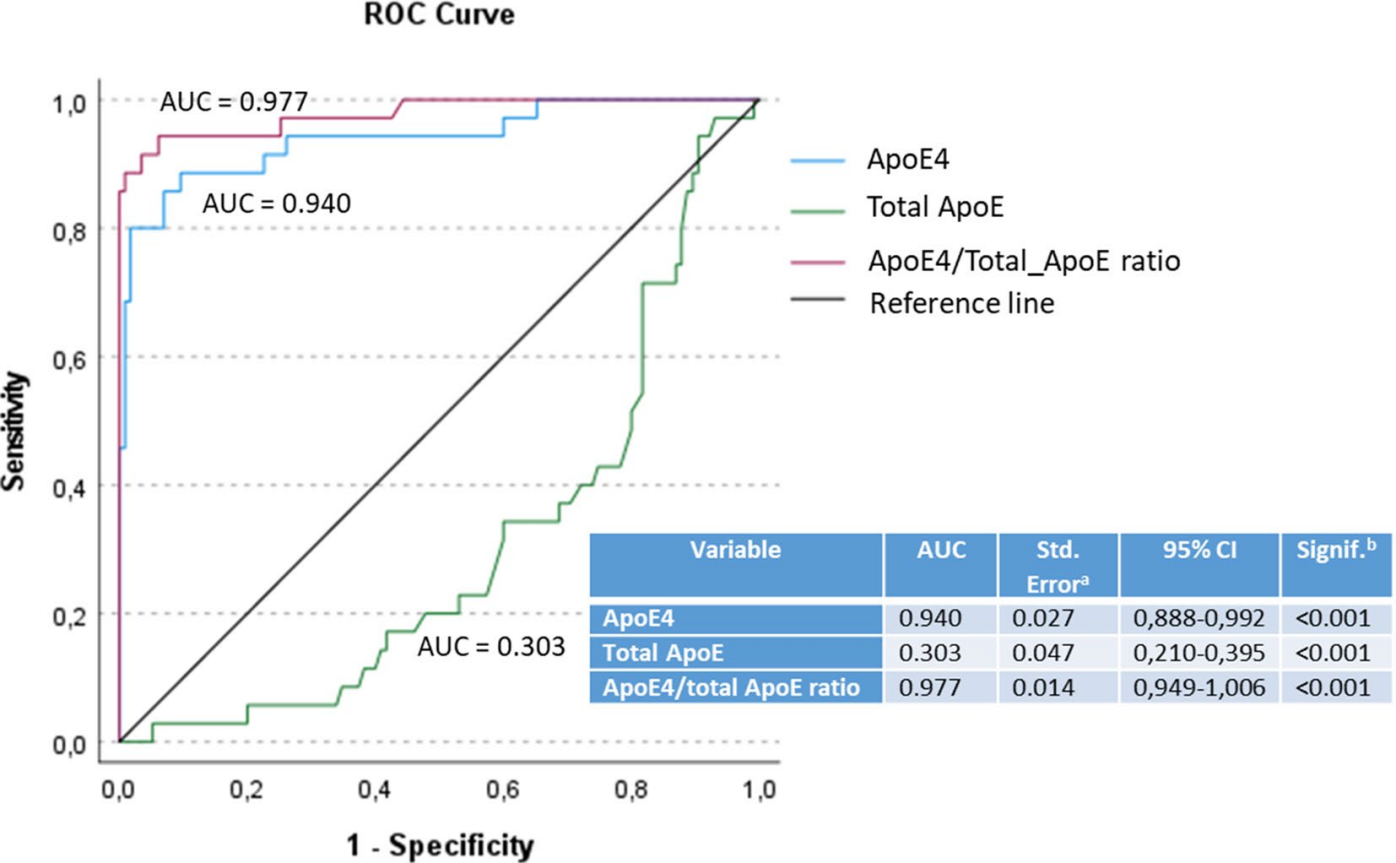
Receiver Operating Characteristic (ROC) curves comparing the discriminatory capability of plasma ApoE4 concentration, total ApoE concentration, and the ApoE4/total ApoE ratio to discriminate between *APOE* ε4 heterozygotes and ε4/ε4 homozygotes. The area under the curve (AUC) is indicated for each variable, with corresponding statistical values in the inset table. The ApoE4/total ApoE ratio yielded the highest discriminative power (AUC = 0.977), followed by ApoE4 alone (AUC = 0.940), whereas total ApoE had poor discriminative capacity (AUC = 0.303). All curves were statistically different from the null hypothesis reference line (AUC = 0.5), indicating significant discriminatory power (*p* < 0.001)

**Fig. 3:**
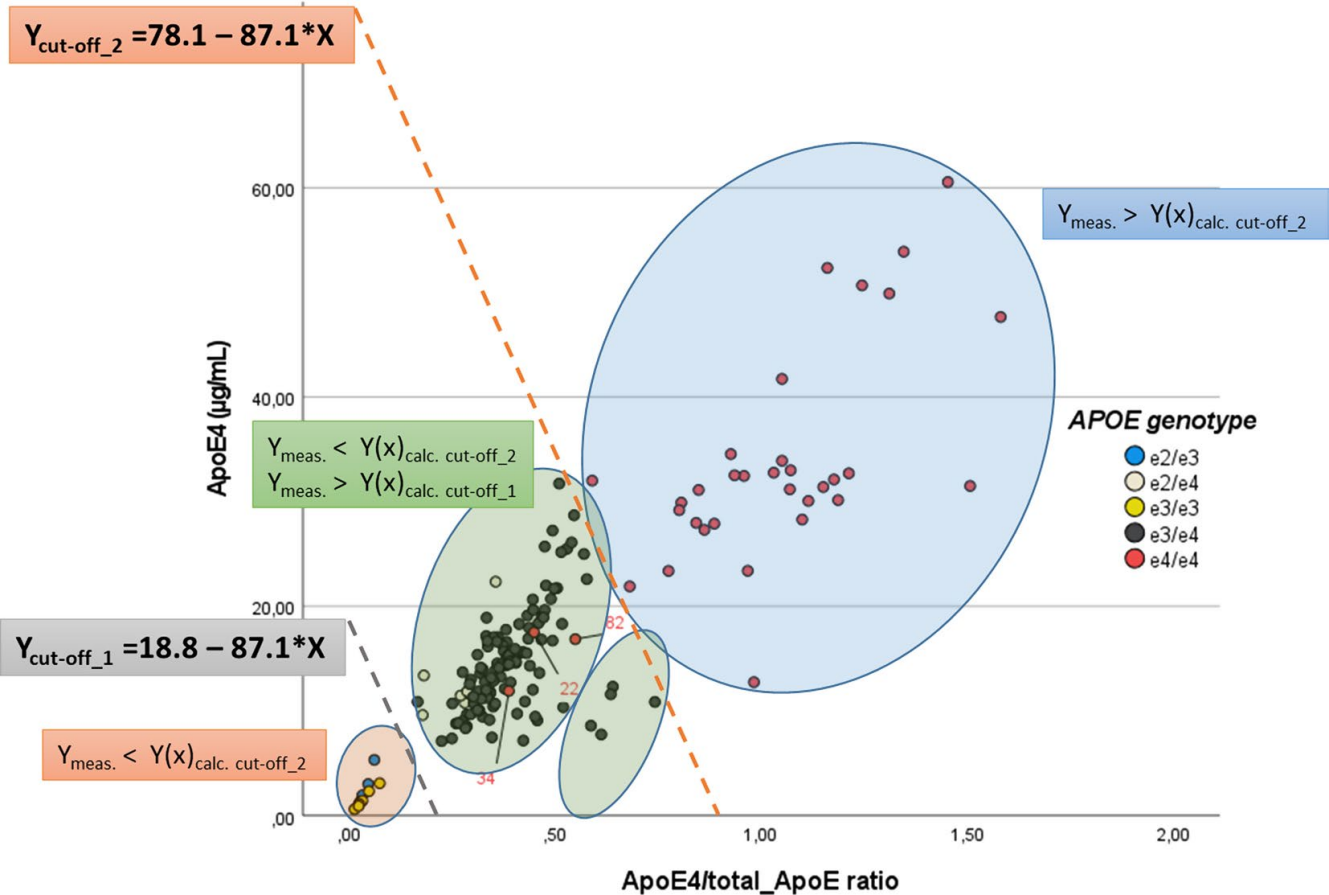
Scatter plot of ApoE4 concentration versus the ApoE4/total ApoE ratio across different *APOE* genotypes. Each dot represents an individual, color-coded according to *APOE* genotype: ε2/ε3 (blue), ε2/ε4 (light grey), ε3/ε3 (yellow), ε3/ε4 (dark green), and ε4/ε4 (red). Ellipses colors indicate classification areas for the different genotypic groups: *APOE* e4 non-carriers (orange), *APOE* ε4 heterozygous (green), and *APOE* ε4/ε4 homozygous (blue). The plot exhibits distinct clustering patterns, with ε4/ε4 individuals displaying markedly higher ApoE4 levels and ApoE4/total ApoE ratios, while ε3/ε3 and ε2/ε3 individuals cluster at lower values. Additionally, to standardize the classification of *APOE* genotype groups, we applied a two-threshold model using parallel linear equations that divided the biomarker space into three informative zones. The upper boundary (y = 78.1–87.1·x) delineates a region containing the majority of *APOE* ε4/ε4 homozygotes, achieving 91.4% sensitivity and 100% specificity for this group. The lower boundary (y = 18.8–87.1·x) excludes all ε4 carriers, providing 100% sensitivity and specificity for identifying ε4 non-carriers. The intermediate zone between both thresholds corresponds predominantly to ε4 heterozygotes but also includes three ε4/ε4 homozygotes with low ApoE4 expression

Graphical representations of the data were generated using GraphPad Prism v10.4 (Figs. 1 and 3, and Supplementary Fig. 1) or IBM SPSS Statistics software v25 (Fig. 2).

## Results

We developed a quantitative turbidimetric assay, e4Quant, for measuring ApoE4 levels in plasma by incorporating a four-point calibration curve and defined quality controls. Calibrators were prepared by spiking known amounts of recombinant ApoE4 protein into a pooled plasma sample from *APOE* ε4 non-carriers. These calibrators were used to generate a standard curve for quantitative analysis. Turbidimetric optical density values from test plasma samples were interpolated against this curve to determine ApoE4 concentrations.

Using the e4Quant test, we analyzed ApoE4 in 160 plasma samples from 10 *APOE* ε4 non-carriers, 115 *APOE* ε4 heterozygous individuals, and 35 *APOE* ε4 homozygous individuals (Table 1). The data for individual *APOE* genotypes, along with total ApoE, ApoE4 levels, and the ApoE4/Total ApoE ratio for each sample, are provided in Supplementary Table 4.

**Table 1:** ApoE4 and total ApoE levels by *APOE* ε4 genotype

APOE ε4 status	*n*	ApoE4 ± SD (µg/ml)	Total ApoE ± SD (µg/ml)	Ratio ApoE4/Total ApoE ± SD
ε4 non-carriers	10	2.12 ± 1.41	51.83 ± 14.40	0.04 ± 0.02
ε4 heterozygotes	115	14.70 ± 5.00	38.44 ± 10.37	0.39 ± 0.10
ε4 homozygotes	35	32.48 ± 11.21	32.79 ± 7.43	1.00 ± 0.32

### Discriminatory ability of the e4Quant test

As expected, ApoE4 levels measured by e4Quant showed different mean values based on the number of *APOE* ε4 alleles expressed (Table 1; Fig. 1 left). The comparison of ApoE4 levels across the three *APOE* genotype groups (non-carriers, ε4 heterozygotes, and ε4 homozygotes) yielded statistically significant differences with p-values < 0.001, indicating robust group separation (Fig. 1 left).

To enhance discrimination among *APOE* ε4 genotype groups, a two-step classification algorithm based on dual cut-off values was evaluated using data from Fig. 1. In the first step, a lower threshold of 6.5 µg/mL was applied to achieve 100% sensitivity for excluding *APOE* ε4 non-carriers. In the second step, a higher threshold of 31.9 µg/mL, determined by ROC analysis, yielded 100% specificity for identifying *APOE* ε4 homozygotes, though with a sensitivity of 46%. This approach defines a grey zone between the two cut-offs (6.5–31.9 µg/mL), where additional genotyping or normalization using total ApoE measurements (see below) is recommended to differentiate ε4 heterozygotes from homozygotes.

The discriminatory ability of the e4Quant test was also studied using ROC analysis, yielding 100% sensitivity and 100% specificity for differentiating *APOE* ε4 carriers from non-carriers, and 88.6% sensitivity and 90.4% specificity (AUC = 0.940) for distinguishing *APOE* ε4 homozygous from heterozygous individuals (cut-off = 21.86 µg/mL) (Figs. 1 left and 2).

### Normalization of ApoE4 to total ApoE levels and discriminatory ability

To test whether normalizing ApoE4 concentration (Fig. 1, left) to total ApoE levels (Fig. 1, center) could improve the discriminatory accuracy of the e4Quant test, we calculated the ApoE4/total ApoE ratio for each sample (Fig. 1, right) by estimating total ApoE with a commercially available test.

Regarding the ApoE4 levels, we employed a two-step classification strategy for the ApoE4/total ApoE ratio to distinguish *APOE* ε4 non-carriers, *APOE* ε4 heterozygotes, and homozygotes (Fig. 1, right). The first cut-off, set at a ratio of 0.12, achieved 100% sensitivity and specificity for ruling out *APOE* ε4 non-carriers. The second cut-off, at a ratio of 0.759, provided 100% specificity for identifying homozygotes, with a corresponding sensitivity of 86%. As with the ApoE4 concentration-based approach alone, this dual cut-off strategy creates a grey zone between the two thresholds (0.12–0.7585), where further testing (such as genotyping) may be warranted to achieve definitive classification.

ROC analysis of the ApoE4/total ApoE ratio determined that the optimal cut-off of 0.548 yielded an improved sensitivity of 94.3% and specificity of 93.9% for distinguishing homozygous from heterozygous *APOE* ε4 carriers (Figs. 1, right, and 2).

To further enhance classification accuracy, we graphically represented ApoE4 levels versus the ApoE4/total ApoE ratio (Fig. 3). Visual inspection of the data distribution revealed that most samples clustered in distinct regions according to the number of *APOE* ε4 alleles. Thus, this approach allowed the correct classification of 10 out of 10 *APOE* ε4 non-carriers (orange ellipse), 115 out of 115 *APOE* ε4 heterozygotes (green ellipses), and 32 out of 35 *APOE* ε4/ε4 homozygotes (blue ellipse) (Fig. 3). This represents a specificity of 100% and a sensitivity of 91.4% in discriminating *APOE* ε4/ε4 homozygotes from heterozygotes. Only 3 out of 35 *APOE* ε4/ε4 homozygous samples (#22, #34, and #82) were misclassified as heterozygous samples (Fig. 3 and Supplementary Table 4).

In addition to this subjective boundaries classification, to add robustness to the analysis, we applied a two-threshold model using parallel linear equations that divided the biomarker space into three informative zones (Fig. 3). By using the equation y = a– 87.1·x, where *x* is the ApoE4/total ApoE ratio and *y* the ApoE4 concentration (µg/mL), two cut-off lines were defined: an upper boundary (cut-off_2: y = 78.1–87.1·x) and a lower boundary (cut-off_1: y = 18.8–87.1·x). Samples below the lower boundary excluded all ε4 carriers, yielding 100% sensitivity and specificity for ruling out the presence of the ε4 allele. Samples above the upper cut-off were classified as likely *APOE* ε4/ε4 homozygotes, capturing 32 out of 35 of homozygous individuals with 91.4% sensitivity and 100% specificity. The intermediate zone between the two cut-offs contained all of ε4 heterozygotes and three low-expressing homozygotes (Fig. 3).

### Analysis of binding affinity of ApoE4 to polystyrene and detection

In order to analyze the binding affinity of ApoE4 to the blocked polystyrene surfaces such as the beads used for turbidimetric assays, we performed an ELISA with increasing amounts of recombinant ApoE4, using for detection either a specific antibody against the ApoE4 isoform or a non-isoform-specific anti-ApoE antibody. This analyses revealed an apparent affinity constant for the interaction ApoE4-polystyrene around 20 nM, but with markedly different quantitation curves (Supplementary Fig. 1). The saturation curve of ApoE4, obtained with the isoform-specific anti-ApoE4 antibody, displayed a rectangular hyperbolic shape, consistent with a one-site binding model. Fitting the data to an equation of *Specific Binding with Hill Slope* equation yielded a Hill slope close to 1, supporting the absence of cooperativity. In contrast, the saturation curve generated using the non-isoform-specific antibody exhibited a sigmoidal shape, indicating a cooperative binding (Supplementary Fig. 1). When fitted to the same model, the curve yielded a Hill slope of 2.65, suggesting that this antibody recognizes multiple binding sites on ApoE with positive cooperativity (Supplementary Fig. 1).

## Discussion

In this study, we present the characterization of a novel non-genetic assay, the **e4Quant test** (Biocross SL, Spain), designed for the accurate quantification of ApoE4 levels in plasma and for distinguishing between *APOE* ε4 homozygous and heterozygous individuals. This assay represents an advancement of the previously developed proprietary test, **e4Risk**^®^ (Biocross SL, Spain), which was initially designed to detect the presence of ApoE4 protein in human plasma using high-throughput clinical chemistry analyzers [27, 28].

Using the e4Quant assay, we quantified ApoE4 levels in 160 human plasma samples, comprising *APOE* ε4 non-carriers, and both *APOE* ε4 heterozygous and homozygous individuals. As expected, mean ApoE4 concentrations differed markedly across genotypes, with *APOE* ε4 non-carriers showing levels consistent with the background of the technique, and levels for the *APOE* ε4 homozygous individuals that were twice those of the heterozygous individuals. Statistically significant differences among all three groups confirmed the assay’s ability to discriminate between genotype classes (see Table 1; Fig. 1, left).

Total ApoE levels showed a decreasing trend with an increasing number of *APOE* ε4 alleles (Fig. 1, center and Table 1), in line with previous results [31, 32]. Notably, extensive population-based studies have consistently reported lower plasma ApoE levels in individuals carrying one or two ε4 alleles, and have linked these reduced levels to an increased risk of dementia and mortality, independently of genotype [4, 31, 33–35].

The e4Quant test by itself provides a perfect differentiation of *APOE* ε4 carriers from *APOE* ε4 non-carriers and a good discriminatory capability to distinguish *APOE* ε4 homozygous from *APOE* ε4 heterozygous (sens = 88.6%, spec = 90.4%) to differentiate *APOE* e4 homozygous from heterozygous (Fig. 1, left). By using a two-step classification algorithm based on dual cut-off values (6.5 and 31.9 µg/mL), the e4Quant assay by itself was able to correctly rule out all *APOE* ε4 non-carriers, and detect specifically a set of *APOE* ε4 homozygotes (sens = 0.46) (Fig. 1, left). By normalizing ApoE4 values to total ApoE, we were able to discriminate *APOE* ε4/ε4 homozygotes from heterozygotes with a specificity of 100% and a sensitivity of 91.4% (Figs. 1, right, 2, and 3). By applying a two-step classification algorithm for the ApoE4/total ApoE ratio with cut-off ratio values of 0.12 and 0.759, we were able to correctly assess all APOE ε4 non-carriers and detect specifically a set of *APOE* ε4 homozygotes (sens = 0.86) (Fig. 1, right). This strategy for either the standalone e4Quant assay or the ApoE4/total ApoE ratio prioritizes both maximal sensitivity to ensure appropriate treatment access (e.g., for anti-amyloid therapy) and maximal specificity to minimize unnecessary risk, particularly in the context of treatment-related side effects.

Further analysis by graphical representation of the ApoE4 levels versus the ApoE4/total ApoE ratio (Fig. 3), followed by a two-threshold model, allowed the correct classification of all *APOE* ε4 non-carriers, as well as all ε4 heterozygotes. Interestingly, as seen in Fig. 3, although all the heterozygous samples can be correctly assigned, five samples fall within a separate region with relatively low levels of ApoE4. Only 3 out of 35 *APOE* ε4/ε4 homozygous samples were misclassified as heterozygous samples (spec = 1; sens = 0.914), primarily due to a low ApoE4/total ApoE ratio in samples with low ApoE4 levels (Fig. 3 and Supplementary Table 4). This approach allowed the meaningful stratification of individuals based on quantitative ApoE4 measurements and ApoE4/total ApoE ratio, offering a practical tool for genotype inference in both clinical screening and research contexts where rapid classification is needed. Visual inspection of the samples ruled out lipemia, hemolysis, and other artifacts. In any case, it remains to be determined whether the low ApoE4/total ApoE ratio detected in a few samples has physiological or pathological implications. Moreover, while further research, particularly longitudinal studies in diverse populations, is essential to validate these findings, recent evidence suggest that *APOE* ε4 homozygosity may represent a distinct genetic form of AD with near-full penetrance rather than merely a risk factor [36].

However, it remains to be determined whether *APOE* ε4/ε4 homozygous individuals with decreased circulating ApoE4 levels, as detected in this study, may have a reduced risk of developing AD. This observation points to underlying biological variability within the homozygous group, potentially stemming from impaired hepatic synthesis or secretion, altered *APOE* mRNA expression, or increased peripheral clearance and degradation of the ApoE4 protein [37, 38]. It is important to recognize that plasma ApoE originates primarily from the liver and reflects peripheral metabolism, whereas central nervous system (CNS) ApoE is independently synthesized by astrocytes and microglia [39, 40]. These two pools are compartmentalized by the blood–brain barrier, and thus, plasma ApoE4 levels may not directly correlate with ApoE4 concentrations in the brain. From a functional standpoint, low circulating ApoE4 in homozygotes could impair peripheral lipid transport and reduce systemic amyloid-β clearance, potentially exacerbating AD pathology. Conversely, lower ApoE4 levels might attenuate its known pro-inflammatory and neurotoxic effects, suggesting a possible protective effect in some individuals. This paradox highlights the complex role of ApoE4 and supports the existence of biologically distinct subgroups among ε4 homozygotes with potentially divergent clinical outcomes. Clinically, these findings underscore the importance of careful interpretation of ApoE4 concentrations in homozygous individuals. In the context of anti-amyloid therapies, such as lecanemab, reduced ApoE4 levels could influence both treatment efficacy and the risk of ARIA. Therefore, integrating ApoE4 quantification with genotypic information may improve patient stratification and support more individualized therapeutic decision-making [41].

A key consideration in interpreting our results is the composition of the study population, which was not intended to reflect the genotype distribution of the general population. Instead, the cohort was deliberately enriched for *APOE* ε4 carriers—particularly ε4 homozygotes—to enable a focused evaluation of the e4Quant assay’s ability to discriminate between ε4 homozygotes and heterozygotes. While this enrichment introduces a deviation from natural prevalence and may influence reported sensitivity and specificity values, it was necessary to support the primary goal of this study: the establishment of robust, quantitative thresholds for genotype discrimination. In this sense, it is also relevant that although no quantitative comparison was conducted between platforms, the inclusion of data from Atellica and Alinity (two of the most widely used platforms in the in vitro diagnostics field) provides critical evidence that the e4Quant assay yields consistent and reliable results beyond the Kroma Plus system.

Opposite to the ApoE total test, which is based on a capture anti-ApoE antibody, the e4Quant test relies on the specific interaction of ApoE with the polystyrene beads, the principal component of the turbidimetry assay. Thus, the aberrant behavior of the three *APOE* ε4/ε4 homozygous samples, in theory, could be associated with a different performance of the tests for the quantitation of ApoE4 and total ApoE at relatively low concentrations. To test this hypothesis, we performed quantitative ELISAs for ApoE using the procedure described [27], which is based on the binding of ApoE to a polystyrene ELISA plate. Notably, these ELISA experiments revealed distinct binding behaviors depending on the detection antibody: the 4E4 ApoE4-specific antibody followed a non-cooperative, one-site binding model, whereas the pan-ApoE antibody exhibited cooperative binding, suggesting multivalent interactions (Supplementary Fig. 1).

These findings may have significant implications for the interpretation of ApoE quantification assays. The distinct binding behaviors of the isoform-specific and non-isoform-specific antibodies (non-cooperative versus cooperative, respectively) may introduce substantial discrepancies in signal detection, particularly at low ApoE4 concentrations. The sigmoidal response curve of the non-isoform-specific antibody, characterized by a Hill slope greater than 1, indicates a minimal signal at low antigen concentrations, with a sharp increase occurring only after the threshold is surpassed. This cooperative behavior may lead to overestimating total ApoE relative to ApoE4 in samples with low expression levels, thus artificially lowering the ApoE4/total ApoE ratio. Such a phenomenon could explain the three misclassified homozygous cases in our dataset, all of which showed low levels of both ApoE4 and total ApoE, clustering within the region associated with *APOE* ε4 heterozygotes (see Fig. 3). If this interpretation holds, reanalyzing borderline samples using a greater volume of plasma (thereby increasing the total amount of both ApoE4 and total ApoE) would likely enhance the assay’s discriminatory power and reduce the risk of misclassification.

In light of the new biological disease-modifying therapies targeting amyloid-beta (Aβ), such as aducanumab [12, 42], lecanemab [43, 44], and donanemab [11, 45], determining the *APOE* ε4 carrier status has become an essential component of the diagnostic and therapeutic work-up in AD [46, 47]. Thus, the availability of a rapid, accessible, and highly accurate method for detecting ApoE4 concentration enables clinicians to efficiently estimate the *APOE* ε4 status, thereby providing neurologists with relevant information to guide both diagnosis and treatment decisions in patients with suspected or confirmed AD.

It is well established that the *APOE* gene, particularly the ε4 allele, is the most potent genetic risk factor for late-onset AD [6, 7, 9, 36] and is also associated with an increased risk and severity of adverse effects, specifically ARIA, in patients undergoing anti-amyloid therapies, such as monoclonal antibodies against amyloid-beta (Aβ) [10]. This risk is particularly elevated in *APOE* ε4/ε4 homozygotes who are treated with amyloid-targeting therapies [11, 44, 48, 49]. Consequently, reliable identification of *APOE* ε4 status is essential for optimizing both the safety and effectiveness of emerging therapeutic approaches.

Interestingly, the *APOE* e4 allele appears to induce common pathological mechanisms that contribute to both increased AD risk and heightened susceptibility to ARIA during anti-amyloid therapy. Thus, the *APOE* ε4 allele seems to impair the clearance of brain amyloid-β, leading to the accumulation of amyloid plaques and cerebral amyloid angiopathy [50, 51], which contributes to blood-brain barrier dysfunction [50], induces an exaggerated inflammatory response and neuroinflammation, and interferes with lipid metabolism and neuronal repair [50, 52].

Understanding the genetic influence on ARIA risk can optimize therapeutic outcomes while minimizing potential complications associated with amyloid-targeting interventions, especially those related to *APOE* ε4/ε4 homozygotes. Currently, some clinical trials, such as ALZ-801 (Valiltramiprosate) [53], are already investigating how to treat AD patients carrying *APOE* ε4 alleles by targeting the reduction of ARIA incidence, and thus allowing a broader application of anti-amyloid therapies and potentially benefiting a larger patient population However, these approaches are not yet readily available; therefore, *APOE* genotyping is an essential component of patient risk stratification before initiating anti-amyloid therapy, aiding in personalized treatment decisions and monitoring strategies to mitigate adverse effects [44].

Moreover, knowing the *APOE* ε4 status could be of interest, as individuals with the *APOE* ε4 allele may benefit from tailored lifestyle changes. For example, recent research suggests that specific diets or supplements may help slow cognitive decline in individuals carrying the *APOE* ε4 allele [54–59]. In addition, emerging evidence indicates that *APOE* ε4 carriers may exhibit distinct metabolic and lipid profiles that could be modifiable through personalized nutritional or pharmacological strategies [60].

Besides its role in AD and neurodegeneration, the *APOE* ε4 allele is also a well-established genetic risk factor for cardiovascular disease (CVD), primarily due to its effects on lipid metabolism and inflammation. *ApoE* plays a crucial role in lipoprotein clearance, and individuals carrying the *APOE* ε4 allele exhibit increased levels of low-density lipoprotein (LDL) cholesterol and total cholesterol, predisposing them to atherosclerosis and coronary artery disease [61]. Compared to the more common *APOE* ε3 allele, ε4 is associated with impaired clearance of remnant lipoproteins and increased oxidative stress, further contributing to endothelial dysfunction and vascular inflammation [62]. Additionally, *APOE* ε4 carriers have been reported to exhibit higher carotid intima-media thickness and a greater risk of ischemic stroke, emphasizing its impact on cerebrovascular health [63–65]. Thus, the association between *APOE* ε4 and CVD risk highlights the importance of genetic screening, particularly in populations with a family history of cardiovascular disorders.

Lastly, beyond classification, e4Quant provides additional value by quantifying ApoE4 levels, which may reveal biological heterogeneity within *APOE* ε4 carriers and offer insights into AD risk stratification and ARIA susceptibility. This dual information (genotype status and protein expression) may be particularly useful in identifying individuals at higher or lower risk and guiding their inclusion in prevention trials or early treatment strategies, aligning with the current AD prevention goals strategy [66–69], and supporting efforts to personalize interventions based on ApoE4 levels in *APOE* ε4 carriers [70].

Limitations of the current study include a relatively small cohort with few *APOE* ε4 non-carriers and its cross-sectional design, which limits conclusions on longitudinal changes or treatment effects. Additionally, while the test does not perfectly separate ε4 homozygotes from heterozygotes, the observation of low ApoE4 levels in some homozygotes raises essential questions about their actual disease risk and ARIA susceptibility, questions that warrant further investigation.

## Conclusions

Biocross e4Quant is a simple, cost-effective, and scalable assay for quantifying ApoE4 in plasma based on chemistry analyzers already available in clinical laboratories. It offers a practical alternative to genetic testing or other biochemical approaches. In contrast to methods such as IEF, ELISA, CLEIA and NULISA, which are either labor-intensive or require specialized platforms, not yet widely accessible, e4Quant is immediately deployable in high-throughput clinical settings. It enables both *APOE* ε4 status inference and ApoE4 protein quantitation from the same plasma sample used in routine dementia assessments.

In summary, e4Quant is a practical tool for *APOE* ε4 detection and ApoE4 quantification, providing valuable insights for guiding AD treatments and lifestyle interventions, potentially enhancing their efficacy and safety profiles. Additionally, ApoE4 concentration in *APOE* ε4 carriers may serve as a biomarker for susceptibility to associated pathologies, including AD and cardiovascular conditions. The assay’s accessibility and quantitative output underscore its potential application in both clinical practice and translational research, contributing to the advancement of personalized medicine, and deeper understanding of ApoE biology in AD. Moreover, its ability to stratify ε4 carriers based on protein expression may support clinical triage for anti-amyloid therapies and inclusion criteria in AD prevention trials.

## Electronic supplementary material

Below is the link to the electronic supplementary material.

Supplementary Material 1

## Data Availability

Data is provided within the manuscript or supplementary information files.

## Abbreviations

AD: Alzheimer’s disease
ApoE: Apolipoprotein E
ARIA: Amyloid-Related Imaging Abnormalities
CSF: Cerebrospinal fluid
CLEIA: Chemiluminescent Enzyme Immunoassay
NULISA: Nucleic Acid–Linked Immunosandwich Assay
PETIA: Particle-enhanced immunoturbidimetric assay
ROC curve: Receiver Operating Characteristic curve
