# Supplementary material for: A novel biochemical analysis for ApoE4 quantification in plasma and discrimination of homozygous and heterozygous APOE ε4 carriers
Source: Alzheimers Res Ther. 2025 Jul 15;17:163. doi: 10.1186/s13195-025-01811-w (PMC12265242; doi:10.1186/s13195-025-01811-w)
Supplement: Supplementary file 1 — Supplementary Material 1 [file 13195_2025_1811_MOESM1_ESM.docx]

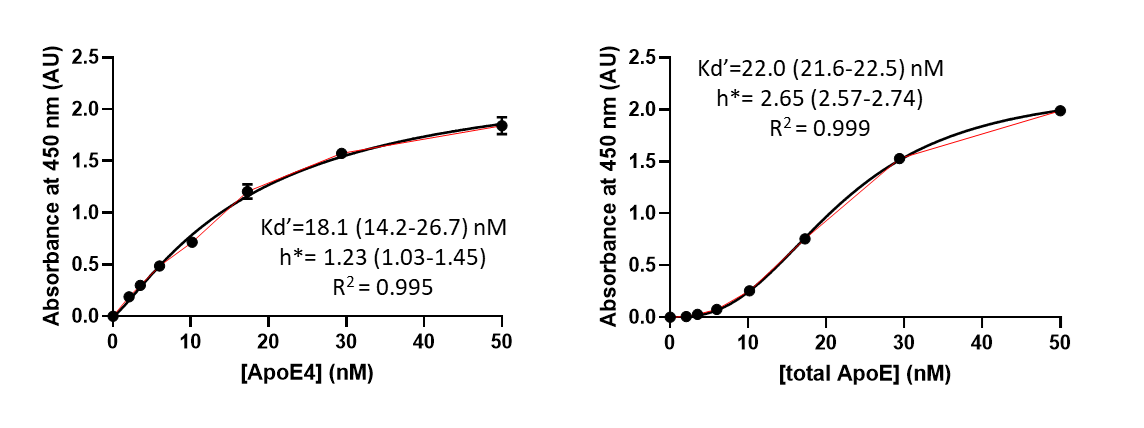


**Suplementary Figure 1.** Saturation binding curves of increasing amounts of ApoE4 bound to polystyrene ELISA plates were detected by a specific anti-ApoE4 antibody (left) or a non-isoform-specific anti-ApoE antibody (right). Fitting (red line) according to the *Specific Binding with Hill Slope* equation (GraphPad Prism 10.4). Model equation: Y = Bmax*X^h/(Kd’^h + X^h). Kd': apparent equilibrium dissociation constant; h. Hill slope.

**Supplementary Table 1. Cross-validation in *Atellica* (Siemens) and *Alinity* (Abbott) platforms of the calibration set**

|  | **Cal 01 (5.32µg/mL)** | | **Cal 02 (9.93µg/mL)** | | **Cal 03 (17.52µg/mL)** | | **Cal 04 (35.43 µg/mL)** | |
| --- | --- | --- | --- | --- | --- | --- | --- | --- |
|  | Siemens | Abbott | Siemens | Abbott | Siemens | Abbott | Siemens | Abbott |
| **Rep 1 (Abs)** | 0.046 | 0.0344 | 0.1702 | 0.1074 | 0.4958 | 0.3287 | 0.8306 | 0.5059 |
| **Rep 2 (Abs)** | 0.0472 | 0.0371 | 0.1738 | 0.1142 | 0.4939 | 0.3089 | 0.8146 | 0.5029 |
| **Rep 3 (Abs)** | 0.0445 | 0.0349 | 0.1757 | 0.1018 | 0.4996 | 0.3175 | 0.8349 | 0.5136 |
| **CV < 10** | 2.95 | 4.03 | 1.61 | 5.76 | 0.58 | 3.12 | 1.29 | 1.09 |

**Supplementary Table 2. Cross-validation in *Atellica* (Siemens) and *Alinity* (Abbott) platforms of low and high controls**

|  | **Low control 6.77µg/mL (5.42–8.12)** | | **High control 9.14 µg/mL (7.32–10.97)** | |
| --- | --- | --- | --- | --- |
|  | Siemens | Abbott | Siemens | Abbott |
| **Result (µg/mL)** | 6.95 | 7.303 | 8.96 | 9.811 |
| **Deviation (%) < 20%** | 2.65 | 7.87 | 1.96 | 6.83 |

**Supplementary Table 3. Cross-validation in *Atellica* (Siemens) and *Alinity* (Abbott) platforms of a set of eight test samples (S#1-S#8)**

| **ID** | ***APOE* genotype** | **Siemens (µg/mL)** | **Abbott (µg/mL)** | **SD** |
| --- | --- | --- | --- | --- |
| S#1 | e3/e3 | 3.65 | 4.11 | **0.33** |
| S#2 | e3/e3 | 3.33 | 3.03 | **0.21** |
| S#3 | e3/e3 | 3.55 | 3.03 | **0.37** |
| S#4 | e3/e3 | < 1.00 | 1.78 | **N/A** |
| S#5 | e3/e3 | 1.63 | < 1.00 | **N/A** |
| S#6 | e3/e4 | 10.65 | 11.66 | **0.71** |
| S#7 | e3/e4 | 8.41 | 8.88 | **0.33** |
| S#8 | e3/e4 | 9.74 | 9.68 | **0.04** |

**Supplementary Table 4.** Demographic data, *APOE* genotypes, ApoE4, and total ApoE levels for the study cohort composed of 35 *APOE* ε4/ε4 homozygous, 115 *APOE* ε4 heterozygous (ε2/ε4 n = 6 and ε3/ε4 n = 109), and 10 *APOE* ε4 non-carriers (ε2/ε3 n = 3 and ε3/ε3 n = 7) individuals.

| Code ID | Age at analysis (y) | Sex | *APOE* haplotype | ApoE4 (µg/mL) | ApoE total (µg/mL) | Ratio ApoE4/total ApoE |
| --- | --- | --- | --- | --- | --- | --- |
| #001 | 37.9 | Female | e3/e4 | 7.2 | 17.0 | 0.42 |
| #002 | 71.4 | Female | e3/e4 | 8.6 | 14.7 | 0.59 |
| #003 | 48.3 | Female | e3/e4 | 12.3 | 19.3 | 0.64 |
| #004 | 67.8 | Female | e3/e4 | 16.6 | 43.4 | 0.38 |
| #005 | 77.8 | Male | e3/e4 | 7.8 | 12.7 | 0.61 |
| #006 | 71.9 | Female | e3/e4 | 10.4 | 19.9 | 0.52 |
| #007 | 53.9 | Female | e3/e4 | 9.1 | 19.9 | 0.46 |
| #008 | 56.9 | Female | e4/e4 | 12.8 | 13.0 | 0.98 |
| #009 | 79.1 | Female | e3/e4 | 10.9 | 25.5 | 0.43 |
| #010 | 74.4 | Female | e3/e4 | 9.5 | 20.9 | 0.45 |
| #011 | 67.5 | Female | e4/e4 | 31.5 | 20.9 | 1.51 |
| #012 | 69.9 | Male | e3/e4 | 9.7 | 33.8 | 0.29 |
| #013 | 68.8 | Female | e3/e4 | 16.3 | 43.7 | 0.37 |
| #014 | 60.0 | Female | e2/e4 | 22.4 | 62.8 | 0.36 |
| #015 | 63.7 | Male | e2/e4 | 10.8 | 38.4 | 0.28 |
| #016 | 76.5 | Female | e3/e4 | 19.1 | 40.7 | 0.47 |
| #017 | 50.9 | Female | e3/e4 | 14.4 | 37.8 | 0.38 |
| #018 | 68.7 | Female | e3/e4 | 13.8 | 39.1 | 0.35 |
| #019 | 52.6 | Male | e3/e4 | 18.4 | 40.1 | 0.46 |
| #020 | 67.6 | Female | e3/e4 | 11.8 | 34.5 | 0.34 |
| #021 | 50.6 | Male | e3/e4 | 14.6 | 40.4 | 0.36 |
| #022 | 58.7 | Female | e4/e4 | 17.5 | 39.1 | 0.45 |
| #023 | 46.5 | Female | e3/e4 | 11.9 | 37.8 | 0.31 |
| #024 | 71.1 | Male | e3/e4 | 31.7 | 62.2 | 0.51 |
| #025 | 55.2 | Female | e3/e4 | 12.0 | 27.0 | 0.45 |
| #026 | 71.7 | Female | e3/e4 | 15.3 | 37.6 | 0.41 |
| #027 | 69.5 | Female | e3/e4 | 18.9 | 56.7 | 0.33 |
| #028 | 55.3 | Male | e3/e4 | 25.5 | 48.3 | 0.53 |
| #029 | 66.5 | Male | e4/e4 | 32.8 | 31.8 | 1.03 |
| #030 | 60.8 | Female | e3/e4 | 25.2 | 48.9 | 0.52 |
| #031 | 78.3 | Female | e3/e4 | 13.0 | 43.9 | 0.30 |
| #032 | 66.9 | Male | e2/e4 | 11.5 | 42.3 | 0.27 |
| #033 | 66.6 | Male | e3/e4 | 16.8 | 36.1 | 0.47 |
| #034 | 68.4 | Female | e4/e4 | 11.9 | 30.8 | 0.39 |
| #035 | 76.7 | Female | e3/e4 | 10.9 | 14.7 | 0.74 |
| #036 | 64.0 | Female | e3/e4 | 7.5 | 21.6 | 0.35 |
| #037 | 68.5 | Female | e3/e4 | 13.6 | 29.5 | 0.46 |
| #038 | 63.2 | Female | e3/e4 | 9.8 | 23.9 | 0.41 |
| #039 | 75.1 | Male | e3/e4 | 11.6 | 18.3 | 0.63 |
| #040 | 83.5 | Male | e3/e4 | 15.6 | 37.1 | 0.42 |
| #041 | 74.8 | Male | e3/e4 | 12.1 | 32.2 | 0.38 |
| #042 | 76.7 | Male | e3/e4 | 15.7 | 41.7 | 0.38 |
| #043 | 71.2 | Female | e4/e4 | 32.1 | 27.3 | 1.18 |
| #044 | 64.0 | Female | e3/e4 | 13.6 | 42.7 | 0.32 |
| #045 | 74.6 | Female | e3/e4 | 9.5 | 29.2 | 0.32 |
| #046 | 69.4 | Male | e3/e4 | 10.8 | 35.5 | 0.30 |
| #047 | 80.2 | Female | e3/e4 | 9.0 | 32.8 | 0.27 |
| #048 | 76.9 | Male | e3/e4 | 26.1 | 48.3 | 0.54 |
| #049 | 71.5 | Male | e3/e4 | 13.3 | 36.1 | 0.37 |
| #050 | 84.3 | Male | e3/e4 | 9.7 | 32.8 | 0.30 |
| #051 | 76.2 | Male | e3/e4 | 9.2 | 26.9 | 0.34 |
| #052 | 79.2 | Female | e3/e4 | 13.7 | 49.6 | 0.28 |
| #053 | 82.7 | Female | e3/e4 | 12.7 | 32.5 | 0.39 |
| #054 | 80.3 | Female | e3/e4 | 17.1 | 47.3 | 0.36 |
| #055 | 78.4 | Male | e3/e4 | 8.5 | 29.8 | 0.29 |
| #056 | 71.5 | Female | e4/e4 | 30.2 | 25.4 | 1.19 |
| #057 | 62.4 | Female | e3/e4 | 17.2 | 51.6 | 0.33 |
| #058 | 78.2 | Female | e3/e4 | 17.1 | 48.7 | 0.35 |
| #059 | 57.1 | Female | e3/e4 | 20.7 | 46.3 | 0.45 |
| #060 | 75.6 | Male | e4/e4 | 31.1 | 36.7 | 0.85 |
| #061 | 55.9 | Male | e4/e4 | 33.9 | 32.3 | 1.05 |
| #062 | 69.9 | Female | e3/e4 | 21.8 | 43.0 | 0.51 |
| #063 | 79.5 | Female | e3/e4 | 14.5 | 39.1 | 0.37 |
| #064 | 80.6 | Female | e3/e4 | 14.0 | 43.0 | 0.32 |
| #065 | 84.2 | Male | e3/e4 | 10.4 | 29.8 | 0.35 |
| #066 | 69.4 | Male | e3/e4 | 15.4 | 49.3 | 0.31 |
| #067 | 75.3 | Female | e3/e4 | 18.3 | 44.4 | 0.41 |
| #068 | 85.3 | Female | e3/e4 | 15.4 | 38.4 | 0.40 |
| #069 | 74.1 | Female | e3/e4 | 17.8 | 46.7 | 0.38 |
| #070 | 76.8 | Female | e3/e4 | 22.0 | 46.1 | 0.48 |
| #071 | 74.0 | Female | e3/e4 | 11.7 | 34.1 | 0.34 |
| #072 | 79.2 | Female | e4/e4 | 52.4 | 45.1 | 1.16 |
| #073 | 78.5 | Female | e3/e4 | 16.2 | 48.0 | 0.34 |
| #074 | 81.2 | Male | e3/e4 | 15.1 | 39.2 | 0.38 |
| #075 | 72.7 | Female | e3/e4 | 16.7 | 46.3 | 0.36 |
| #076 | 76.4 | Male | e3/e4 | 16.7 | 33.9 | 0.49 |
| #077 | 77.6 | Female | e3/e4 | 19.7 | 41.4 | 0.48 |
| #078 | 82.1 | Male | e3/e4 | 12.8 | 38.8 | 0.33 |
| #079 | 66.5 | Male | e3/e4 | 11.6 | 34.8 | 0.33 |
| #080 | 71.6 | Female | e3/e4 | 19.1 | 44.2 | 0.43 |
| #081 | 81.9 | Female | e3/e4 | 16.7 | 49.6 | 0.34 |
| #082 | 74.8 | Female | e4/e4 | 16.9 | 30.8 | 0.55 |
| #083 | 88.7 | Female | e3/e4 | 15.4 | 39.8 | 0.39 |
| #084 | 78.1 | Female | e3/e4 | 19.7 | 44.0 | 0.45 |
| #085 | 78.0 | Female | e3/e4 | 14.2 | 44.2 | 0.32 |
| #086 | 83.2 | Female | e3/e4 | 15.2 | 39.5 | 0.38 |
| #087 | 72.7 | Male | e3/e4 | 14.6 | 36.1 | 0.40 |
| #088 | 78.0 | Female | e3/e4 | 11.3 | 33.6 | 0.34 |
| #089 | 73.8 | Female | e3/e4 | 14.1 | 38.9 | 0.36 |
| #090 | 56.1 | Female | e3/e3 | 0.6 | 46.0 | 0.01 |
| #091 | 70.1 | Male | e3/e4 | 18.0 | 41.1 | 0.44 |
| #092 | 79.9 | Female | e4/e4 | 31.2 | 29.2 | 1.07 |
| #093 | 62.5 | Female | e3/e4 | 15.3 | 39.5 | 0.39 |
| #094 | 63.7 | Male | e3/e3 | 3.1 | 41.1 | 0.08 |
| #095 | 70.9 | Male | e3/e4 | 12.0 | 36.1 | 0.33 |
| #096 | 75.7 | Female | e3/e4 | 27.3 | 55.1 | 0.49 |
| #097 | 61.0 | Male | e2/e3 | 3.0 | 64.2 | 0.05 |
| #098 | 77.7 | Female | e3/e4 | 25.7 | 54.2 | 0.47 |
| #099 | 73.7 | Male | e3/e4 | 11.2 | 34.8 | 0.32 |
| #100 | 78.5 | Male | e3/e4 | 7.1 | 31.7 | 0.22 |
| #101 | 85.8 | Female | e3/e4 | 16.9 | 38.9 | 0.43 |
| #102 | 68.1 | Male | e2/e4 | 11.9 | 41.4 | 0.29 |
| #103 | 73.5 | Female | e4/e4 | 28.3 | 25.8 | 1.10 |
| #104 | 64.5 | Female | e4/e4 | 30.1 | 27.0 | 1.11 |
| #105 | 71.8 | Male | e3/e4 | 8.8 | 33.9 | 0.26 |
| #106 | 71.3 | Female | e3/e3 | 1.3 | 47.7 | 0.03 |
| #107 | 72.7 | Male | e4/e4 | 34.6 | 37.3 | 0.93 |
| #108 | 62.6 | Female | e4/e4 | 31.4 | 27.3 | 1.15 |
| #109 | 74.6 | Female | e3/e4 | 28.7 | 52.6 | 0.55 |
| #110 | 80.7 | Male | e3/e4 | 12.9 | 36.7 | 0.35 |
| #111 | 78.6 | Female | e4/e4 | 28.0 | 33.3 | 0.84 |
| #112 | 71.4 | Female | e4/e4 | 60.6 | 41.7 | 1.45 |
| #113 | 81.2 | Female | e3/e4 | 11.4 | 37.6 | 0.30 |
| #114 | 64.8 | Male | e4/e4 | 23.4 | 24.2 | 0.97 |
| #115 | 86.8 | Female | e3/e4 | 10.1 | 31.7 | 0.32 |
| #116 | 71.3 | Female | e3/e4 | 14.3 | 32.6 | 0.44 |
| #117 | 81.0 | Female | e3/e4 | 10.9 | 65.1 | 0.17 |
| #118 | 78.8 | Male | e3/e4 | 14.8 | 37.6 | 0.39 |
| #119 | 71.1 | Female | e4/e4 | 29.9 | 37.1 | 0.81 |
| #120 | 79.4 | Male | e4/e4 | 32.7 | 27.0 | 1.21 |
| #121 | 72.7 | Female | e4/e4 | 49.9 | 38.1 | 1.31 |
| #122 | 79.6 | Male | e4/e4 | 47.7 | 30.2 | 1.58 |
| #123 | 70.3 | Female | e4/e4 | 23.4 | 30.2 | 0.78 |
| #124 | 60.9 | Female | e3/e4 | 18.3 | 35.4 | 0.52 |
| #125 | 64.5 | Female | e3/e4 | 15.9 | 39.5 | 0.40 |
| #126 | 78.7 | Male | e2/e3 | 5.3 | 85.9 | 0.06 |
| #127 | 63.0 | Female | e3/e4 | 8.8 | 33.3 | 0.27 |
| #128 | 66.0 | Male | e3/e4 | 10.7 | 30.1 | 0.35 |
| #129 | 71.3 | Male | e3/e4 | 14.7 | 36.4 | 0.40 |
| #130 | 63.8 | Male | e3/e4 | 19.0 | 40.1 | 0.47 |
| #131 | 65.7 | Female | e3/e4 | 22.6 | 39.2 | 0.58 |
| #132 | 73.0 | Female | e3/e4 | 25.0 | 43.9 | 0.57 |
| #133 | 56.5 | Female | e4/e4 | 32.5 | 33.9 | 0.96 |
| #134 | 75.1 | Male | e3/e4 | 7.4 | 29.5 | 0.25 |
| #135 | 69.0 | Female | e3/e4 | 10.1 | 32.3 | 0.31 |
| #136 | 86.1 | Female | e3/e4 | 20.7 | 42.3 | 0.49 |
| #137 | 61.0 | Female | e3/e3 | 2.3 | 47.9 | 0.05 |
| #138 | 72.9 | Female | e3/e4 | 21.7 | 43.6 | 0.50 |
| #139 | 73.4 | Female | e4/e4 | 41.7 | 39.7 | 1.05 |
| #140 | 55.7 | Female | e3/e4 | 13.2 | 38.3 | 0.34 |
| #141 | 72.3 | Female | e2/e3 | 2.0 | 59.2 | 0.03 |
| #142 | 64.5 | Male | e4/e4 | 27.3 | 31.7 | 0.86 |
| #143 | 72.4 | Female | e4/e4 | 53.9 | 40.1 | 1.35 |
| #144 | 70.0 | Male | e3/e4 | 15.5 | 36.1 | 0.43 |
| #145 | 61.8 | Female | e3/e3 | 1.4 | 43.6 | 0.03 |
| #146 | 63.8 | Female | e3/e3 | 1.1 | 45.1 | 0.03 |
| #147 | 69.6 | Female | e4/e4 | 50.7 | 40.7 | 1.24 |
| #148 | 76.7 | Male | e3/e3 | 0.9 | 37.6 | 0.02 |
| #149 | 81.2 | Female | e3/e4 | 12.6 | 42.9 | 0.29 |
| #150 | 54.7 | Female | e2/e4 | 9.6 | 53.9 | 0.18 |
| #151 | 60.5 | Female | e3/e4 | 8.4 | 29.5 | 0.28 |
| #152 | 66.0 | Female | e3/e4 | 14.5 | 37.3 | 0.39 |
| #153 | 55.6 | Female | e2/e4 | 13.4 | 73.6 | 0.18 |
| #154 | 55.7 | Female | e3/e4 | 10.7 | 42.6 | 0.25 |
| #155 | 71.7 | Female | e4/e4 | 21.9 | 32.2 | 0.68 |
| #156 | 74.7 | Female | e4/e4 | 32.5 | 34.8 | 0.94 |
| #157 | 61.3 | Female | e4/e4 | 32.0 | 54.3 | 0.59 |
| #158 | 74.4 | Female | e4/e4 | 33.0 | 30.8 | 1.07 |
| #159 | 75.1 | Male | e4/e4 | 27.9 | 31.5 | 0.89 |
| #160 | 72.5 | Female | e4/e4 | 29.2 | 36.4 | 0.80 |
